# Relations between the Crowe classification and the 3D femoral head displacement in patients with developmental dysplasia of the hip

**DOI:** 10.1186/s12891-019-2838-z

**Published:** 2019-11-11

**Authors:** Rongshan Cheng, Henghui Zhang, Willem Alexander Kernkamp, Jingmao Zheng, Kerong Dai, Yifei Yao, Liao Wang, Tsung-Yuan Tsai

**Affiliations:** 1 Shanghai Key Laboratory of Orthopaedic Implants, Department of Orthopaedic Surgery, Shanghai Ninth People’s Hospital, Shanghai Jiao Tong University School of Medicine, School of Biomedical Engineering, Shanghai Jiao Tong University, Shanghai, 200030 China; 2 Guangxi Clinical Research Center for Digital Medicine and 3D Printing, Guigang City People’s Hospital, Guangxi, 537100 China; 3 Engineering Research Center of Clinical Translational Digital Medicine, Ministry of Education of P.R. China, Shanghai, 200030 China

**Keywords:** Developmental dysplasia of the hip, Crowe classification, Three-dimensional displacement of the femoral head, Anterior-posterior, Medial-lateral, Superior-inferior

## Abstract

**Background:**

The purpose of this study was to investigate the relationship between the three dimensional (3D) femoral head displacement in patients with developmental dysplasia of the hip (DDH) and Crowe classification.

**Methods:**

Retrospectively, CT scans of 60 DDH patients and 55 healthy demography-matched healthy control subjects were analyzed. Using the anterior pelvic plane a pelvic anatomic coordinate system was established. The center coordinates of the femoral heads of both the DDH patients and control subjects were quantified relative to the pelvic coordinate system and were mapped proportionally to a representative normal pelvis for comparison.

**Results:**

In the anteroposterior (AP) direction, the center of the femoral head was significantly more anterior in the DDH patients (type I, II, and III, respectively45.0 ± 5.5, 42.9 ± 7.1, and 43.9 ± 4.6 mm) when compared to the controls (50.0 ± 5.2 mm) (*p* < 0.001 for all). In the medial-lateral (ML) direction, the center of the femoral head was significantly more lateral in the DDH patients (type I, II, and III =103.5 ± 8.6, 101.5 ± 6.6, 102.1 ± 11.2 mm) when compared to the controls (87.5 ± 5.1 mm) (*p* < 0.001 for all). In the superior-inferior (SI) direction, the center of the femoral head was significantly more proximal in the DDH patients (type I, II, and III =62.4 ± 7.3, 50.0 ± 6.3, and 43.2 ± 6.6 mm) when compared to the controls (66.0 ± 6.2 mm) (*p* < 0.001 for all).

**Conclusions:**

The severity of DDH using the Crowe classification was related to the degree of the femoral head displacement in the SI direction, but not in the ML or AP directions. By assessing the 3D femoral head displacement in DDH patients, individualized component positioning might benefit surgical outcome.

## Introduction

Total hip arthroplasty (THA) in patients with developmental dysplasia of the hip (DDH) is technically difficult and challenging due to severe deformity and anterolateral bone deficiency [1]. THA, as treatment of DDH, has a higher complication rate and lower patient satisfaction rates when compared to THA performed for primary degenerative osteoarthritis [2]. Malpositioning of the center of rotation (COR) of the hip has been shown to increase risks of postoperative complications, e.g., limb-length discrepancy (LLD) [3], abductor muscle weakness [4], dislocation [5] and aseptic loosening [6]. Appropriate reconstruction of hip COR in THA is essential for restoration of hip function and improvement in clinical outcome in DDH patients [7]. An accurate understanding of the femoral head displacement in DDH patients could aid in improving pre-operative planning and treatment modalities.

Different classification systems have been used to grade the severity of DDH such as the Crowe classification, the Hartofilakidis classification, and the Eftekhar and Kerboul classification [8]. The Crowe classification the most frequently used classification in literature is the Crowe classification [9]. The Crowe classification considers the distance from the femoral head center to the inferior margin of the acetabulum (i.e., the acetabular teardrop) on a plain anterior-posterior (AP) radiograph to categorize the value of femoral head displacement. Thus, the Crowe classification is a two-dimensional (2D) assessment and may, therefore, be unable to reflect 3-dimensional (3D) morphological changes of the femoral head, especially deformities in the AP direction. The 3D femoral head displacement remains unknown, accurate 3D measurement of the femoral head displacement may improve surgical planning of THA and preoperative THA placement in DDH patient which could lead to improved outcomes [10].

We hypothesized that the Crowe classification does not reflect the 3D femoral head displacement in DDH patients. The purpose of this study was to investigate, (1) the 3D femoral head displacement in DDH patients compared to healthy controls, and (2) the association between the 3D femoral head displacement in DDH patients with different Crowe types.

## Methods

### Patients

**Fig. 1:**
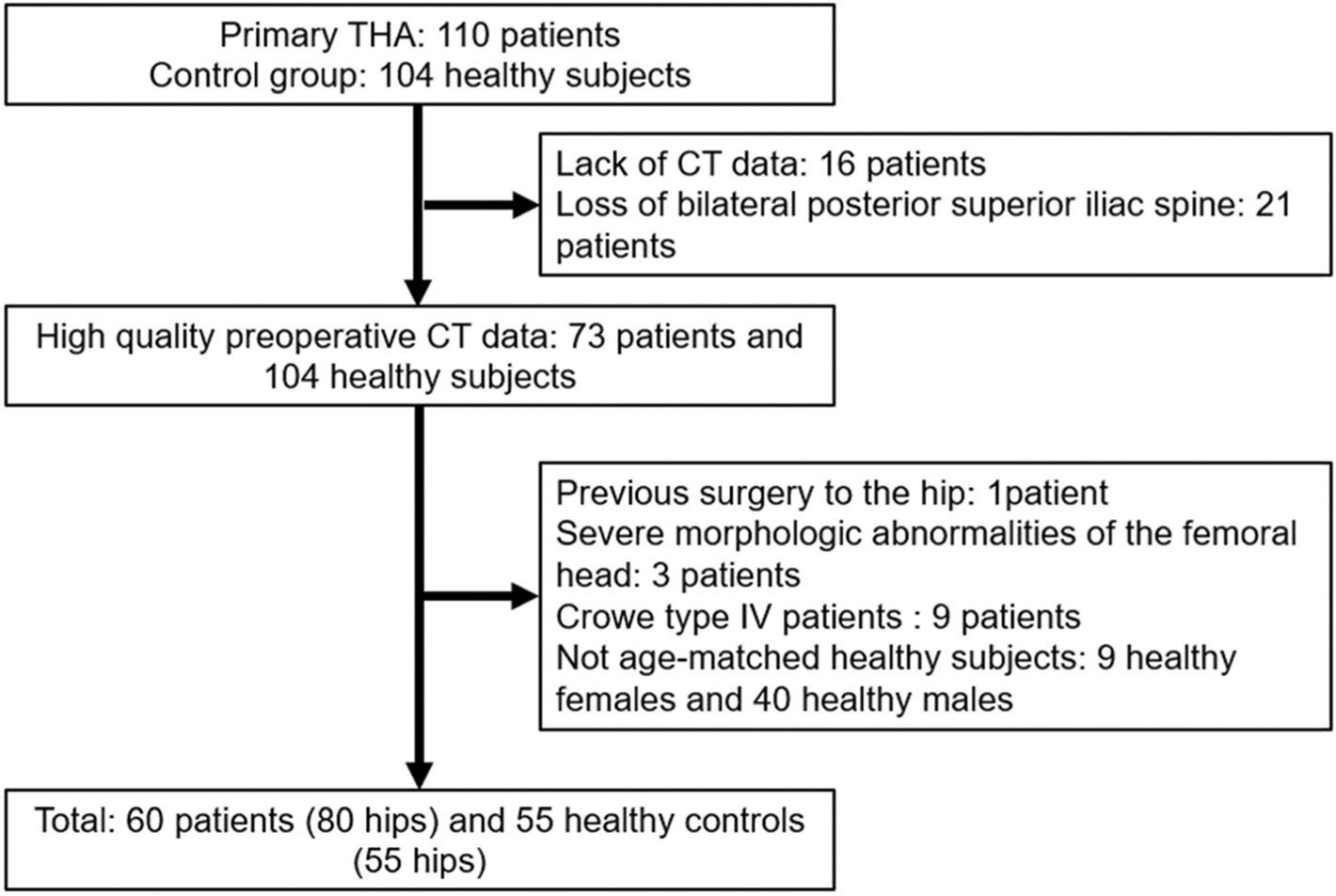
Flow chart diagram of patient selection

The Institutional Review Board at our institute approved this retrospective study. The preoperative computed tomography (CT) scans of 110 DDH patients scheduled for THA (from November 2007 to April 2017) from a single arthroplasty surgeon (ZZ) practice were retrieved as DDH patient group, and 104 high-resolution CT angiography scans of the lower limbs for diagnosis of osteonecrosis in our institution’s database (from October 2011 to January 2015) were considered as healthy control group. The inclusion criteria for DDH patients was a lateral center-edge angle of Wiberg less than 20° [11]. The Crowe classification was determined using an AP radiograph. The exclusion criteria for DDH patients were previous hip surgery or another hip disease, and lack of femoral head making it impossible to measure the femoral heads, e.g., Crowe type IV patients. The inclusion criteria for healthy controls were center-edge angle 25° or more, and a sharp angle less than 45°. The exclusion criteria for healthy controls were surgical treatment of hip disease, hip abnormalities or degenerative changes, and self-reported hip symptoms (Fig. 1). Total of 60 DDH patients and 55 healthy control subjects were included in this study. No statistically significant differences between the DDH (Crowe I-III) patient group and the healthy control group were found in demographic data including age, gender, height, weight, and BMI using one-way analysis of variance (ANOVA) (Table 1).

**Table 1:** Comparison of characteristics between DDH (Crowe I-III) patients and healthy controls

Parameters	Healthy Controls, N = 55hips	Dysplastic, N = 48hips Crow I	Dysplastic, N = 20hips Crow II	Dysplastic, N = 12hips Crow III	P †
Age^a^ (yrs.)	55.8 ± 6.7	59.9 ± 8.8	55.1 ± 11.5	55.6 ± 15.6	0.245
Sex(no.)					0.107
Male	15	6	4	5	
Female	40	23	15	7	
Side(no.)					0.001‡
Right	55	28	14	7	
Left	0	20	6	5	
Height^a^ (cm)	158.8 ± 7.2	159.5 ± 6.2	160.1 ± 6.0	157.8 ± 3.5	0.902
Weight^a^ (kg)	60.1 ± 6.3	58.7 ± 8.4	59.2 ± 6.0	58.8 ± 3.7	0.933
BMI^a^ (kg/m2)	23.8 ± 2.1	23.1 ± 3.2	23.1 ± 2.2	23.6 ± 1.4	0.810
Pelvic width^a^ (cm)	274.1 ± 14.8	267.4 ± 17.0	270.4 ± 17.2	263.6 ± 16.4	0.084
Pelvic height^a^ (cm)	198.9 ± 9.8	199.5 ± 11.8	195.9 ± 11.2	194.1 ± 9.2	0.322
Pelvic depth^a^ (cm)	133.1 ± 10.2	133.5 ± 9.9	132.5 ± 8.9	129.9 ± 6.4	0.706

† P values were obtained by ANOVA or chi-square test for comparisons in the DDH (Crowe I-III) patients and the healthy controls

‡ Significant difference between the DDH (Crowe I-III) patients and the healthy controls at 0.01 level

^a^Values express mean ± SD

### Radiologic evaluations

**Fig. 2:**
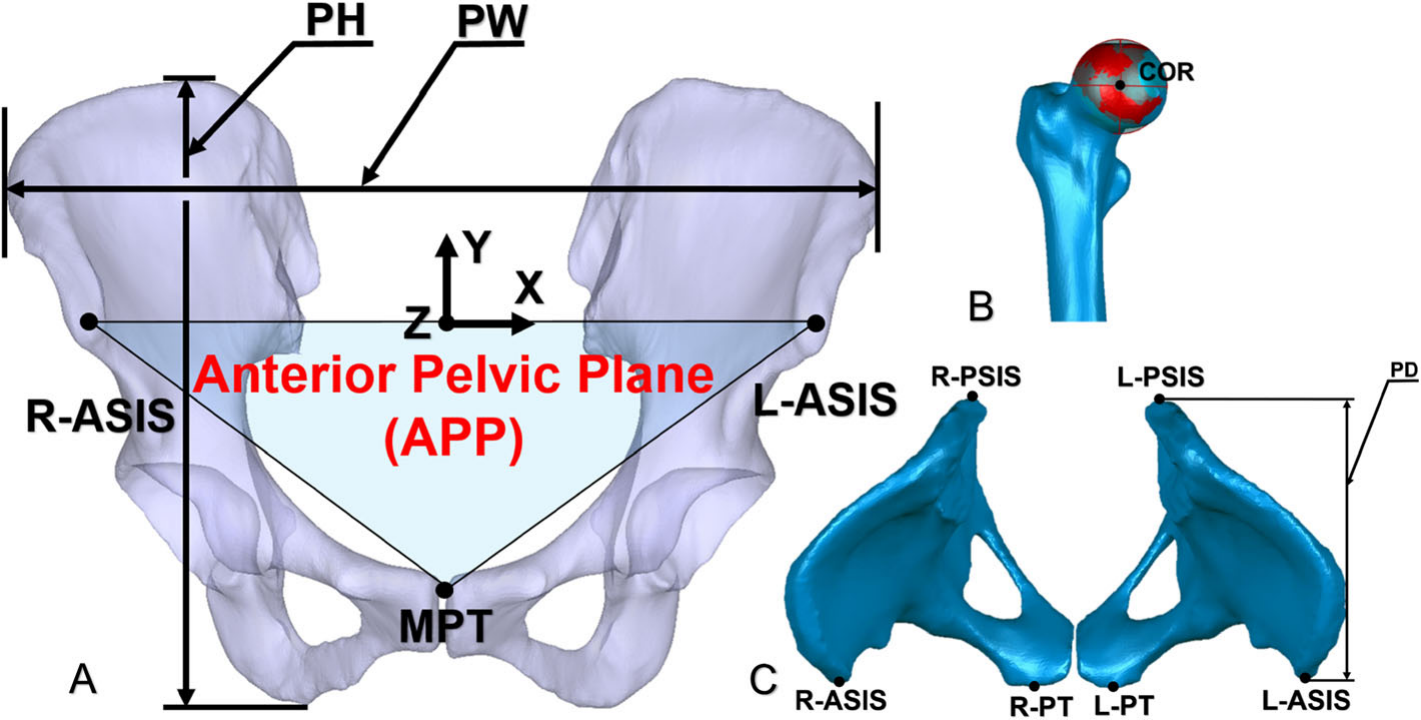
**a** The anterior pelvic plane (APP) was used for the pelvic coordinates, based on the anatomic bony landmarks, including the right anterior superior iliac spine (R-ASIS), the left anterior superior iliac spine (L-ASIS) and the midpoint of the pubic tubercles (MPT). The origin of the pelvic coordinate system was at the mid-point between two ASISs in the X-axis running from the R-ASIS to the L-ASIS. The Y-axis was parallel to the normal vector of the plane formed by two ASISs and the mid-point of PSISs and passing through the origin and the middle of pubic tubercles. The Z-axis was the cross product of the X and Y axes. The pelvic width (PW) and the pelvic height (PH) were noted. **b** The center of rotation (COR) was defined as the centroid of the best sphere (red-covered surface) to the surface of the femoral head (the average of standard deviations (STD) of the best-fit sphere of all femoral heads is < 0.4 mm). **c** Bony landmarks of the pelvis including anterior-superior iliac spines (ASIS), pubic tubercles (PT) and posterior-superior iliac spine (PSIS) were digitized. The pelvic depth (PD) was shown

All CT scans were made from the fifth lumbar vertebra to the distal femur. The CT images of the DDH patients were collected using a 128-slices CT scanner (SOMATOM Definition Flash, Siemens Healthcare, Forchheim, Germany) providing an in-plane resolution of 0.98 mm at 1-mm slice thickness. For the healthy controls, the CT images were acquired using a 64-slice CT scanner (Philips Medical Systems, Cleveland, Ohio, USA) providing an in-plane resolution of 0.68 mm at 2-mm slice thickness. The CT scans were then imported into Amira 6.7 (Thermo Fisher Scientific, Waltham, MA, USA) for the reconstruction of 3D surface models of the preoperative pelvis and femur. The 3D surface models of the pelvis and femur were then imported to a custom-made script (MATLAB, The Mathworks Inc., Natick, MA) for further data analysis (Fig. 2a). The center of the femoral heads of the DDH patients and healthy control subjects were defined as the centroid of a best fit 3D sphere to the surface of the femoral head (Fig. 2b). Bony landmarks of the pelvis, including bilateral anterior and posterior superior iliac spines (ASIS and PSIS) and pubic tubercles (PT), were digitized on the 3D bony model for determination of anatomic pelvic coordinate system (Fig. 2c).

### Measurement of the femoral head center

To standardize the geometric measurements, the pelvic coordinate system of each subject was established as the reference using the anterior pelvic plane (APP) [12] (Fig. 2a). According to the methods described by Dandachli et al. [13], pelvic width (PW), pelvic height (PH) and pelvic depth (PD) were defined (Fig. 2a and c). The femoral head center coordinates were measured regarding the origin of the pelvic coordinate system in the pelvic coordinate system. The femoral head center coordinates were then mapped proportionally to a representative normal size pelvis. All the cases with involvement of the left hip were mirrored to the right side for consistent comparisons between groups.

### Statistical analysis

All the continuous data in this study were normally distributed and were expressed as means ± standard deviations. An independent sample t-test was used to compare the difference of the femoral head coordinates in 3D between the DDH patients and the healthy controls. A one-way ANOVA was used to determine whether there was a statistically significant difference in the 3D femoral head center among the DDH patients with different Crowe types. The post hoc multiple comparisons were conducted by the Student-Newman-Keuls test. The significant statistical level (α) was set at 0.05. Statistical analyses were performed using SPSS (IBM SPSS Statistics, Version 24, IBM Corp., Armonk, NY, USA). The statistical power analysis for t-test or F-tests was calculated with G*power software 3.1.9 (Franz Faul, Christian-Albrechts-Universität Kiel, Kiel, Germany).

In order to assess the intraobserver and interobserver variations, all measurements were repeated in a blinded manner by two researchers (CRS and ZJM), who made the measurements twice at a one-month interval without knowing the first results. The interclass correlation coefficient (ICC) was calculated to evaluate intraobserver and interobserver reliabilities.

## Results

The ICC demonstrated excellent intraobserver (0.92–0.96) and interobserver (0.91–0.94) reliabilities for the femoral head center measurements. The power analyses indicated that our sample size has adequate power (β =0.94) to detect the effects. No statistically significant differences in the pelvic dimensions (PW, PH, and PD) were found between the DDH (Crowe I-III) patients and the healthy controls (Table 1).

**Fig. 3:**
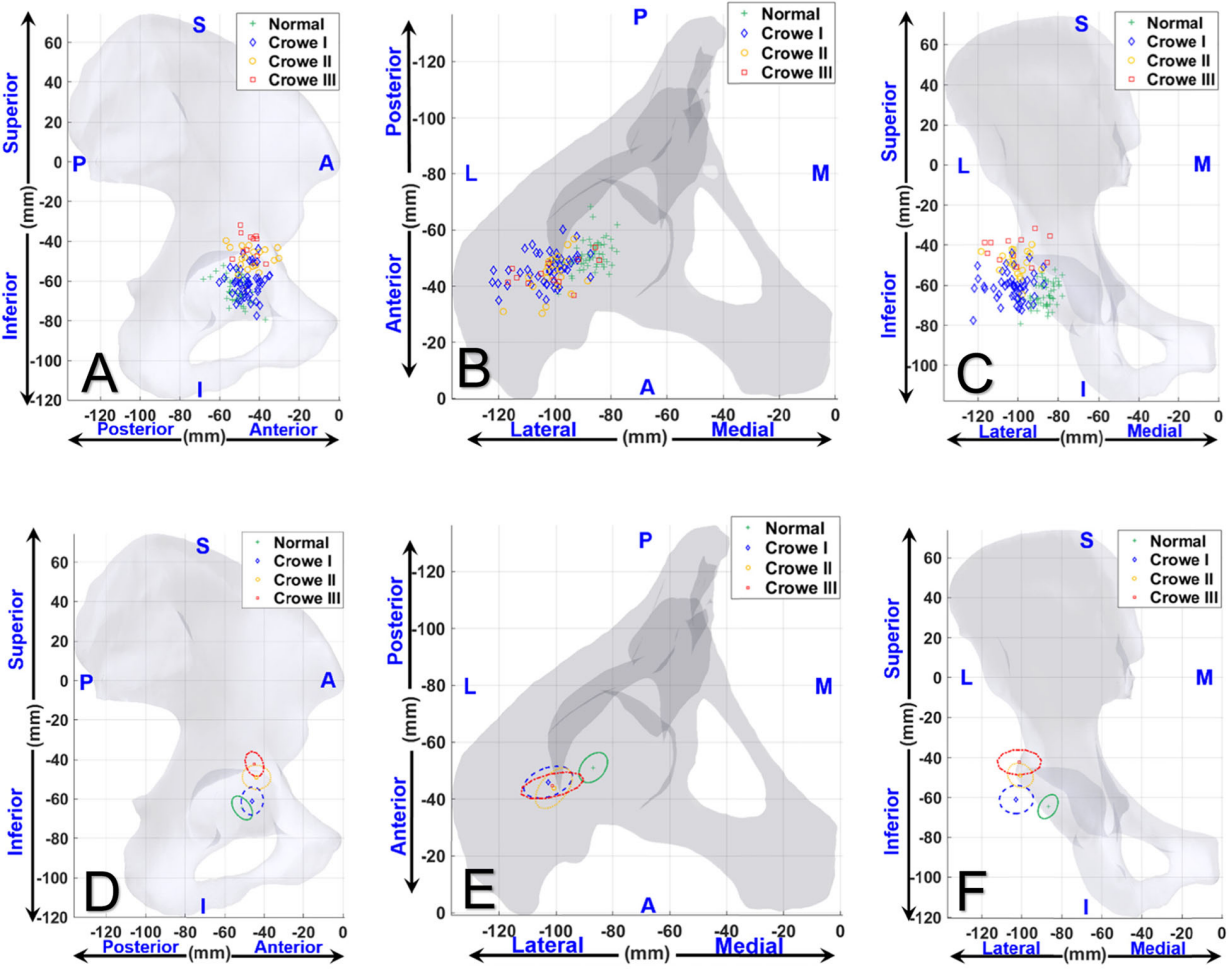
A representative 3D pelvis model and the distribution of 3D displacement of the femoral head centers of the DDH (Crowe I-III) and the healthy controls groups. The distributions (**a**, **b** and **c**) and the standard deviational ellipses (**d**, **e**, and **f**) of 3D displacement of the femoral head centers were shown from the AP, ML, and SI directions. The Crowe classification did not correspond to the degree of the femoral head 3D displacement in patients with DDH. Markers ‘+’ (green),’◊’ (blue),’○’ (yellow), and’□’ (read) represented normal, Crowe type I DDH group, Crowe type II DDH group, and Crowe type III DDH group, respectively

In the anterior-posterior (AP) direction, the center of the femoral heads of the DDH patients were significantly (*p* < 0.001) more anterior (type I, 45.0 ± 5.5 mm [mean ± SD]; type II, 42.9 ± 7.1 mm; type III, 43.9 ± 4.6 mm) t when compared to the healthy controls (50.0 ± 5.2 mm, Table 2, Fig. 3a&d). The Crowe type II group showed the largest average anterior displacement followed by type III group and type I, respectively. No statistically significant differences (*p* = 0.653) of the femoral head position in the AP direction were found among the Crowe I, II and III groups.

**Table 2:** The comparison of the femoral head center location between the DDH (Crowe I-III) group and the healthy control group with all the data mapped proportionally to a representative normal pelvis (the pelvic width = 272.1 mm, the pelvic height = 194.5 mm and the pelvic depth = 136.1 mm). The comparison of the 3D displacement of the femoral heads in DDH patients of different Crowe types relative to the healthy control group. The femoral head center location of the DDH group is significantly more anterior, superior, and lateral than those of the healthy control group. The Crowe classification corresponded to the degree of the femoral head displacement in the SI and 3D distance, while not reflecting the degree of displacement in the ML and SI directions

Displacement	Media-lateral^a^ (mm)	Superior-inferior^a^ (mm)	Anterior-posterior^a^ (mm)	3D distance^a^ (mm)
Healthy Control Group	87.5(5.1; 86.1–88.9)	66.0(6.2; 64.4–67.7)	50.0(5.2; 48.6–51.4)	
Crowe I	103.5(8.6; 101.0–106.0)^b^	62.4(7.3; 60.3–64.5)^bc^	45.0(5.5; 43.4–46.6)^b^	19.5(8.3; 17.1–21.9)^d^
Crowe II	101.5(6.6; 98.5–104.6)^b^	50.0(6.3; 47.1–53.0)^bc^	42.9(7.1; 39.6–46.2)^b^	24.1(7.3; 20.6–27.5)^d^
Crowe III	102.1(11.2; 95.0–109.2)^b^	43.2(6.6; 39.1–47.4)^bc^	43.9(4.6; 41.0–46.8)^b^	29.9(7.5; 25.1–34.7)^d^

^a^ Values express mean (SD; 95%CI)

^b^ Significant differences between the DDH (Crowe I-III) group and the healthy control group at 0.05 level

^c^ Significant differences among the DDH (Crowe I-II-III) group at 0.05 level

^d^ Significant differences among the DDH (Crowe I-II-III) group at 0.05 level

In the medial-lateral (ML) direction, the center of the femoral heads of the DDH patients were significantly (*p* < 0.001) more lateral (type I, 103.5 ± 8.6 mm; type II, 101.5 ± 6.6 mm; type III, 102.1 ± 11.2 mm) when compared to the healthy controls (87.5 ± 5.1 mm, Table 2, Fig. 3b&e). The Crowe type I group showed the largest average lateral displacement followed by type III and type II, respectively. No statistically significant differences (*p* = 0.385) of the femoral head position in the ML direction were found among the Crowe I, II or III groups.

In the superior-inferior (SI) direction, the center of the femoral heads of the DDH patients were significantly (*p* < 0.001) more proximal (type I, 62.4 ± 7.3 mm, type II, 50.0 ± 7.0 mm, type III, 43.2 ± 7.5 mm) when compared to the healthy controls (66.0 ± 6.2 mm). The Crowe type III group had the greatest superior displacement, followed by type II and type I, respectively. Statistically significant differences with one-way ANOVA in the SI direction were found among the Crowe I, II or III groups (F = 47.9, *p* = 3.0 × 10^− 14^, Table 2, Fig. 3c&f).

In the 3D direction, the displacements of the femoral heads in DDH patients relative to the healthy controls were 19.5 ± 8.3 mm, 24.0 ± 7.3 mm and 29.9 ± 7.5 mm for Crowe type I, II and III, respectively. The Crowe type III group has the largest 3D displacement, followed by type II and type I, respectively. Statistically significant differences using one-way ANOVA in the 3D direction were found among the Crowe I, II or III groups (F = 8.8, *p* = 3.5 × 10^− 4^, Table 2).

## Discussion

The most important finding of this study was that the Crowe classification was unable to describe the femoral head displacement of DDH patients. The severity of DDH using the Crowe classification was related to the degree of the femoral head displacement in the SI direction, but not in the ML or AP directions.

Several authors have reported that anterior dislocation of the hip joint can cause insufficient anterior acetabular coverage of the femoral head, and may influence the preoperative planning, surgical procedure, and the functional outcome after THA for DDH patients [9, 14, 15]. In this study, our data indicated that the Crowe type II DDH patients have the most severe anterior displacement of the femoral head, followed by the type III and I, respectively. Argenson et al. showed that Crowe II dysplastic patients have the greatest amount of femoral anteversion [16], and Akiyama et al. reported that femoral anteversion correlated with anterior acetabular coverage and bone deficiency in patients with DDH [17]. Therefore, the degree of femoral head displacement in the AP direction may be an important factor to further improve the THA placement and the restoration of anterior femoral coverage, especially for Crowe type II patients.

The severity of DDH using the Crowe classification cannot be described as the femoral head displacement of DDH patients in the ML direction. Previous studies showed that the lateral translation of the COR after THA changes the bodyweight lever arm, which increases joint reaction force, decreases the abductor muscle efficiency and increases acetabular component loosening rates [4, 7]. Delp et al. reported that a 2 cm lateral displacement of the femoral head increased the abductor moment arm by 20%, the moment by 40% and the force by 26% [18]. Wang et al. reported that the Crowe type I group has the greatest dislocation rate [19]. Our data indicated that the femoral head of DDH patients with Crowe type I, II or III has an on average 1.5 cm more lateral translation than that of the healthy controls. We further found that the degree of femoral head lateral displacement in DDH patients with Crowe type I was the most severe, followed by type III and II, respectively. Therefore, DDH patients with Crowe type I may be more vulnerable to a large abnormal abductor muscles force than the other DDH patients. Furthermore, Meermans et al. reported that the medial displacement of COR would directly influence limb length and should be taken care in order to avoid limb length discrepancy [1]. Accurate assessment of the femoral head ML displacement in DDH patients for THA surgical planning might help surgeons to optimize joint reaction force, abductor muscle force and limb length for the better postoperative outcome.

Our data showed that the maximum displacement of the femoral head occurred in SI direction, which is in line with previous reports using the Crowe classification [9]. In addition, the femoral head displacement of DDH patients in the SI direction was the only dimension that was not significantly different from the Crowe classification. Previously it was reported that limb-length discrepancies can be improved by correct placement of the femoral head in the SI direction during THA [20]. Our data indicated that the degree of displacement of Crowe type I-III of DDH patients was significantly different from the healthy controls in the SI direction. These findings confirmed the rationale of the traditional Crowe classification theory supporting that the Crowe type III group has the greatest superior displacement, followed by type II and I, respectively [9]. Moreover, previous studies have related to limb-length discrepancy after THA to poor patient satisfaction, low back pain, gait disorders and femoral head dislocation [21–23]. Our results suggested that surgeons may be cautious when planning THA in DDH patients, the center of the femoral head in Crowe type III patients, especially in the SI direction can aid in optimizing the leg length for better postoperative patient outcomes.

Determination of the anatomical femoral head center is a crucial step and can be difficult when planning THA. Trousdale et al. reported that the thickness of the medial aspect of the acetabulum in DDH patients was increased, causing the femoral head being lateralized [24]. Bernasek et al. reported that Crowe type I patients had the lowest mean acetabular volume than other Crowe type groups [25]. Based upon our own experience, albeit anecdotical, in Crowe type I patients osteophytes are mostly located at the inner wall of the acetabulum, which may cause the femoral head to move laterally, causing lateral displacement of COR. During surgery, if the COR of the hip cannot be restored to the normal state, the COR after THA will be more lateral than that of the healthy controls, which ultimately can affect the abductor muscle efficiency. Therefore, 3D femoral head displacement assessment in DDH patients may help to improve surgical planning and joint reconstruction.

## Limitations

This study has several limitations. First, the sample size of Crowe type II and III was relatively small due to its low prevalence. However, the statistical power analysis in our study showed that the current sample size would provide 94% power to detect the difference. Additional studies with a larger sample size might be more helpful. Second, as DDH in men is relatively uncommon, a separate assessment of the femoral head center location in men and women could be further analyzed in the future. Third, the measurement of the femoral head center might be affected by the deformity of the femoral head. The high ICCs demonstrated excellent reliability of our measurements. Lastly, our study was limited to Chinese patients. Therefore, our results may not apply to other racial or ethnic groups.

## Conclusions

The center of the femoral heads of DDH patients was significantly more anterior, superior, and lateral than those of the healthy controls. The severity of the DDH using the Crowe classification was related to the degree of the femoral head displacement in the SI direction. However, the Crowe classification cannot reflect the femoral head displacement in the ML and AP directions. The 3D comparative analysis of the femoral head centers in DDH patients and healthy controls provided further insight into the 3D femoral head displacement caused by DDH. These data may help surgeons to further improve preoperative planning and restoration of the hip function for DDH patients.

## Data Availability

The data that support the findings of this study are available from [Shanghai Ninth People’s Hospital, China] but restrictions apply to the availability of these data, which were used under license for the current study, and so are not publicly available. Data are however available from the authors upon reasonable request and with permission of [Shanghai Ninth People’s Hospital, China].

## Abbreviations

2D: Two-dimensional
3D: Three-dimensional
ANOVA: Analysis of variance
AP: Anterior-posterior
APP: Anterior pelvic plane
ASIS and PSIS: Anterior and posterior superior iliac spines
COR: Center of rotation
CT: Computed tomography
DDH: Developmental dysplasia of the hip
ICC: Interclass correlation coefficient
L-ASIS: Left anterior superior iliac spine
LLD: Limb-length discrepancy
ML: Medial-lateral
MPT: Midpoint of the pubic tubercles
PD: Pelvic depth
PH: Pelvic height
PT: Pubic tubercles
PW: Pelvic width
R-ASIS: Right anterior superior iliac spine
SI: Superior-inferior
STD: Standard deviations
THA: Total hip arthroplasty
